# Immunological effects of shift work in healthcare workers

**DOI:** 10.1038/s41598-019-54816-5

**Published:** 2019-12-03

**Authors:** Bette Loef, Nening M. Nanlohy, Ronald H. J. Jacobi, Chantal van de Ven, Rob Mariman, Allard J. van der Beek, Karin I. Proper, Debbie van Baarle

**Affiliations:** 1 Centre for Nutrition, Prevention and Health Services, National Institute for Public Health and the Environment, Bilthoven, The Netherlands; 2 Department of Public and Occupational Health, Amsterdam UMC, Vrije Universiteit Amsterdam, Amsterdam Public Health research institute, Amsterdam, The Netherlands; 3 Centre for Immunology of Infectious Diseases and Vaccines, National Institute for Public Health and the Environment, Bilthoven, The Netherlands; 4 Clinical Operations, Julius Clinical, Zeist, The Netherlands; 5 Department of Immunology, Laboratory for Translational Immunology, University Medical Centre Utrecht, Utrecht University, Utrecht, The Netherlands

**Keywords:** Epidemiology, Immunology

## Abstract

The immune system potentially plays an important mechanistic role in the relation between shift work and adverse health effects. To better understand the immunological effects of shift work, we compared numbers and functionality of immune cells between night-shift and non-shift workers. Blood samples were collected from 254 night-shift and 57 non-shift workers employed in hospitals. Absolute numbers of monocytes, granulocytes, lymphocytes, and T cell subsets were assessed. As read out of immune function, monocyte cytokine production and proliferative capacity of CD4 and CD8 T cells in response to various stimuli were analysed. The mean number of monocytes was 1.15 (95%-CI = 1.05–1.26) times higher in night-shift than in non-shift workers. Furthermore, night-shift workers who worked night shifts in the past three days had a higher mean number of lymphocytes (B = 1.12 (95%-CI = 1.01–1.26)), T cells (B = 1.16 (95%-CI = 1.03–1.31)), and CD8 T cells (B = 1.23 (95%-CI = 1.05–1.45)) compared to non-shift workers. No differences in functional parameters of monocytes and lymphocytes were observed. The differences in numbers of monocytes and T cells suggest that chronic exposure to night-shift work as well as recent night-shift work may influence the immune status of healthcare workers. This knowledge could be relevant for preventive initiatives in night-shift workers, such as timing of vaccination.

## Introduction

Many biological functions in the human body follow a circadian rhythm^1^. Professions involving night shifts require persons to work and sleep at times that conflict with this rhythm. This may result in circadian rhythm disruption and disturbed sleep, which have been proposed as possible sources of health problems associated with shift work^2,3^. Currently, shift work has been linked to an increased risk of cardiovascular, metabolic, and infectious diseases^4–8^.

The physiological mechanisms that connect shift work to these diseases are still not fully understood. It is thought that the immune system may be affected by shift work, and this may subsequently be associated with cardiovascular disease and infection^9–11^. For example, activation of proinflammatory responses of the immune system caused by disturbances in circadian rhythms and sleep may be linked with cardiovascular disease risk^11^. Furthermore, previous studies have indicated that the adaptive and innate immune system display circadian rhythms, and disruption of these immune responses may enhance infection susceptibility^10,12^. Correspondingly, the first studies on shift work and infection susceptibility in humans report a higher incidence and severity of (respiratory) infections in shift workers compared to non-shift workers^6,13–15^.

Immune responses that are under influence of the circadian rhythm, and may therefore be affected by disruption of this rhythm, include, among others, rhythms of leukocytes, phagocytosis, cytokine production, and proliferative responses to antigens^12^. Some studies have reported a higher number of lymphocytes or leukocytes in shift workers^16–21^, which has been suggested to reflect enhanced inflammation and to be associated with increased disease risk^16–21^, while other studies did not find these differences^11,22,23^. With respect to function of immune cells, a study among a small group of workers indicated that proliferative responses were significantly depressed in rotating shift workers^24^, but a more recent study reported no differences in proliferative responses between shift and non-shift workers^23^. Because of the few available studies that show mixed results, more in depth research on the relation between shift work and immune cell distribution and function is needed. Specifically, research using state of the art immune cell analysis based on direct visualization of immune cell numbers and functions through flow cytometry will contribute to this need.

The aim of the present study was to examine the relation between night-shift work and disturbances in immune cell counts and functions. Therefore, we compared numbers of monocytes, granulocytes, lymphocytes, and T cell subsets between night-shift and non-shift workers. As a read out of immune function, we compared monocyte cytokine production and proliferative T cell responses to different stimuli between night-shift and non-shift workers.

## Methods

### Study design

The present study is part of the Klokwerk + study that aims to study the effects of (night) shift work on infection susceptibility and body weight, and the mechanisms underlying these health effects^25,26^. Participants were healthcare workers aged 18–65 years, recruited from different hospitals in the Netherlands. At baseline, participants completed a questionnaire about demographics, shift work, lifestyle, and health. At the follow-up measurement, which took place after approximately six months, blood samples were collected from a subsample of the participants (i.e. all participants who were present at the follow-up measurement and were available for blood sample collection in the morning hours), and participants completed a follow-up questionnaire. Approval of the current study was obtained from the institutional review board of the University Medical Centre Utrecht, The Netherlands (study protocol number 16-044/D, NL56022.041.16). Informed consent was obtained from all participants. The study was carried out in accordance with the standards set by the latest revision of the Declaration of Helsinki.

### Measures

#### Night-shift work

Participants were divided into two groups based on their shift work status reported in the baseline and follow-up questionnaire. Night-shift workers worked rotating shifts (rotating between day, evening, night, and/or sleep shifts) including night shifts (shifts between 00:00–06:00 hours). Non-shift workers did not work rotating shifts and/or night shifts for at least six months before the baseline measurement. The population of night-shift workers was further categorized into groups based on frequency (1-2/3-4/≥5 night shifts/month) and duration (<10/10-19/≥20 years) of night-shift work. To determine exposure to recent night-shift work (yes vs. no), the type of shifts participants worked in the three days prior to blood sample collection was asked.

#### Numbers of immune cells: trucount analysis

All blood samples were collected in the morning between 08:00–13:00 hours. On the same day, absolute numbers of immune cells were determined in whole blood using Trucount tubes (Becton Dickinson, Fullerton, CA). Whole blood samples were incubated with antibodies in Trucount tubes using the following antibodies: CD3-FITC, CD45-PerCP, CD56-PE, CD45RO-PECy7, CD8-APC, CD27-APCeFluor780, CD19-BV421, and CD4-BV510. Erythrocytes were lysed with FACS lysing solution (BD). Samples were acquired on a FACS (fluorescence-activated cell sorting) Fortessa X20 and ≥25,000 CD3+ T cells were acquired. Results were analysed using FlowJo V10 (FlowJo company, Ashland, OR). Monocytes, granulocytes, and lymphocytes (CD45+) were gated. Lymphocytes were separated into B cells (CD45+/CD19+), NK cells (CD45+/CD56+), T cells (CD45+/CD3+), and NKT cells (CD45+/CD3+/CD56+). Subsequently, within the CD4+ and CD8+ T cells, fractions of naive (CD27+/CD45RO−), effector memory (CD27−/CD45RO+), central memory (CD27+/CD45RO+), and effector (CD27−/CD45RO−) T cells were determined (See for gating strategy Supplementary Fig. S1). Per cell type, the absolute cell number in the blood samples was calculated by dividing the number of events in the cell gate by the number of events in the bead gate and multiplying this with the number of beads per test divided by the test volume (i.e. 50 μl).

#### Monocyte profiling

Monocytes can be divided into three different subsets (i.e. classical, intermediate, and non-classical monocytes) that perform different functions (i.e. phagocytosis, pro-inflammatory, and patrolling)^27^. To assess the phenotype of the monocytes, PBMC (peripheral blood mononuclear cells) were thawed and first incubated for 5 min with a Fc Receptor Blocking Solution (human Trustain FcX, Biolegend) and then stained with monoclonal antibodies. Thawed PBMC were used, because these analyses were performed at a later stage of the study. To exclude lymphoid cells, antibodies against lineage markers CD3, CD4, CD15, CD19, CD56, and NKp46 were used and after exclusion of doublets and HLA-DR− cells, monocyte subsets were identified according to their expression of CD14 and CD16 (Supplementary Fig. S2).

#### Functions of immune cells: T cell proliferation assays

Within 24 hours, PBMC were isolated using a Ficoll-Hypaque density gradient centrifugation, cryopreserved, and stored at −135 °C. For the proliferation assays, PBMC were thawed and stained with Celltrace violet (Molecular Probes)^28^. Cells were cultured in RPMI-1640 Medium supplemented with 1% penicillin/streptomycin, and 10% human AB serum. A negative control (medium alone), a positive control (0.05 µg/ml αCD3 and 2 µg/ml αCD28), and five different (super)antigens and mitogens were used to stimulate the PBMC. The following stimuli were used: Tetanus toxoid (TT, 0.3 Lf/ml), Super CEFX pool (sCEFX, 1 µg/ml, containing Coxsackievirus B4, Human adenovirus 5, Human herpesvirus 1–6, Human papillomavirus, JC polyomavirus, Measles virus, Rubella virus, Vaccinia virus, Clostridium tetani, Influenza A virus, Helicobacter pylori, and Toxoplasma gondii), Phytohaemagglutinin (PHA, 0.5 µg/ml), Staphylococcus enterotoxin B (SEB, 0.05 µg/ml), and Super EFX pool (sEFX, 1 µg/ml, similar pool as sCEFX but without Cytomegalovirus (CMV)). The stimuli are named in order of priority, meaning that if there were inadequate numbers of PBMC (i.e. approximately 1–2 * 10^6^ PBMC per stimulus) available to use all stimuli, the last named stimulus was omitted first. The cells were cultured at 37 °C in a humidified atmosphere containing 5% CO_2_ and after 7 days stained with the following antibodies: CD8-FITC, CD3-AlexaFluor700, Fixable Viability Stain-780, Celltrace violet, CD27-BV786, CD45RO-PE, and CD4-BUV395. Celltrace violet was used to label cells in order to trace multiple generations of cells based on dye dilution. Samples were acquired on a FACS Fortessa X20 and FlowJo V10 software was used to analyse the data. T cells were selected from lymphocytes based on expression of CD3. For the negative control and every stimulus, percentages of proliferating CD4 and CD8 T cells were determined by identifying the percentage of cells with celltrace dye dilution (representing all cells that divided) based on scatters of CD4 and CD8 against celltrace violet as indicated in Supplementary Fig. S3. Next, the percentage of proliferating cells cultured with medium (i.e. no stimulation/negative control) was subtracted from the percentage of proliferating cells after stimulation to adjust for the fact that some proliferation might have occurred even without exposure to a stimulus. These outcome measures were then used to determine, for every stimulus separately, whether participants expressed T cell proliferation (yes vs. no), and for the participants who expressed T cell proliferation (i.e. the responders), the magnitude of this response.

#### Proinflammatory cytokines and chemokines

Proinflammatory cytokines and chemokines (IL-8/IP-10/Eotaxin/TARC/MCP-1/RANTES/MIP-1α/MIG/ENA-78/MIP-3a/GROα/I-TAC/MIP-1β/IL-1β/IFN-α2/IFN-γ/TNF-α/IL-6/IL-10/IL-12 p70/IL-17A/IL-18/IL-23/IL-33) in serum and in supernatant of TLR-stimulated PBMC (TLR 1–9 kit, Invivogen) were quantified using a human inflammation panel and a human proinflammatory chemokines panel bead-based immunoassay (LEGENDplex, Biolegend, San Diego) according to the manufacturer’s instructions. For measuring cytokines in supernatant of the T cell proliferation assays at day 1 and day 6, a human Thelper Cytokine panel was used (IL-5/IL-13/IL-2/IL-6/IL-9/IL-10/IFN-γ/TNF-α/IL-17A/IL-17F/IL-4/IL-21/IL-22).

#### Covariates

Age, gender, CMV status (positive vs. negative, determined with Luminex assays), occupation (nurse vs. other healthcare worker), educational level, general perceived health (very good/excellent vs. bad/moderate/good), smoking, influenza vaccination status (did vs. did not receive the seasonal influenza vaccination), and self-reported recent infection in the past two weeks (yes vs. no) were included as covariates. Participants were also asked to indicate whether they were a morning, evening, or intermediate chronotype.

### Statistical analysis

#### Numbers of immune cells: trucount analysis

Numbers of immune cells were expressed as cells/µl. The independent-samples t-test for normally distributed outcomes and the Mann-Whitney U-test for non-normally distributed outcomes were used to compare numbers of cells between night-shift and non-shift workers. Linear mixed model regression analysis was performed on the log-transformed outcomes to adjust for covariates. For reasons of consistency, it was decided to log-transform the data of all outcomes, because half of the outcomes did not follow a normal distribution. In the mixed models, hospital of employment and date of blood sample collection were used as random effects to adjust for correlation between the same batches^29^. Analyses were repeated stratified by recent night-shift work (past three days), frequency (1-2/3-4/≥ 5 night shifts/month) and duration (<10/10-19/≥ 20 years) of night-shift work, and by chronotype of night-shift workers (morning/evening/intermediate chronotype), consistently using non-shift workers as reference group. Analyses were adjusted for all covariates.

#### Functions of immune cells: T cell proliferation assays

Proportions of night-shift and non-shift workers who expressed T cell proliferation as a response to the different stimuli were compared using the chi-square test. Response data (yes: >0% vs. no: 0% stimulus-specific T cell proliferation) was further analysed using logistic regression analysis. Among the responders, percentages of proliferating CD4 and CD8 T cells were compared between night-shift and non-shift workers using the Mann-Whitney U test, separately for the different stimuli. The distribution of the percentages of proliferating CD4 and CD8 cells followed a positively skewed distribution, and transformation of data was not able to adjust for this. Therefore, logistic regression analysis was performed on the outcomes that were dichotomized based on the median. Due to the smaller sample size, the T cell proliferation analyses were only adjusted for CMV status and occupation. Since the assays with the different stimuli have the same outcome measure and aim to answer the same research question, an analysis combining the results of the different assays into one analysis was also conducted. To this end, logistic Generalized Estimating Equations (GEE) analysis was used taking into account the correlation between repeated observations within a subject. Percentages of proliferating CD4 and CD8 T cells were used as the dependent variables.

#### Proinflammatory cytokines and chemokines

Cytokine and chemokine concentrations (in pg/ml) in serum and after stimulation of PBMC were compared between night-shift and non-shift workers using the Mann-Whitney U-test and linear mixed model regression analysis as described above.

Analyses were conducted using IBM SPSS Statistics, V.24.0 (IBM Corp, New York), Stata/SE, V.14.2 (StataCorp LP, College Station), and GraphPad Prism, V.7.04 (GraphPad Software Inc., San Diego).

## Results

### Study population

**Figure 1:**
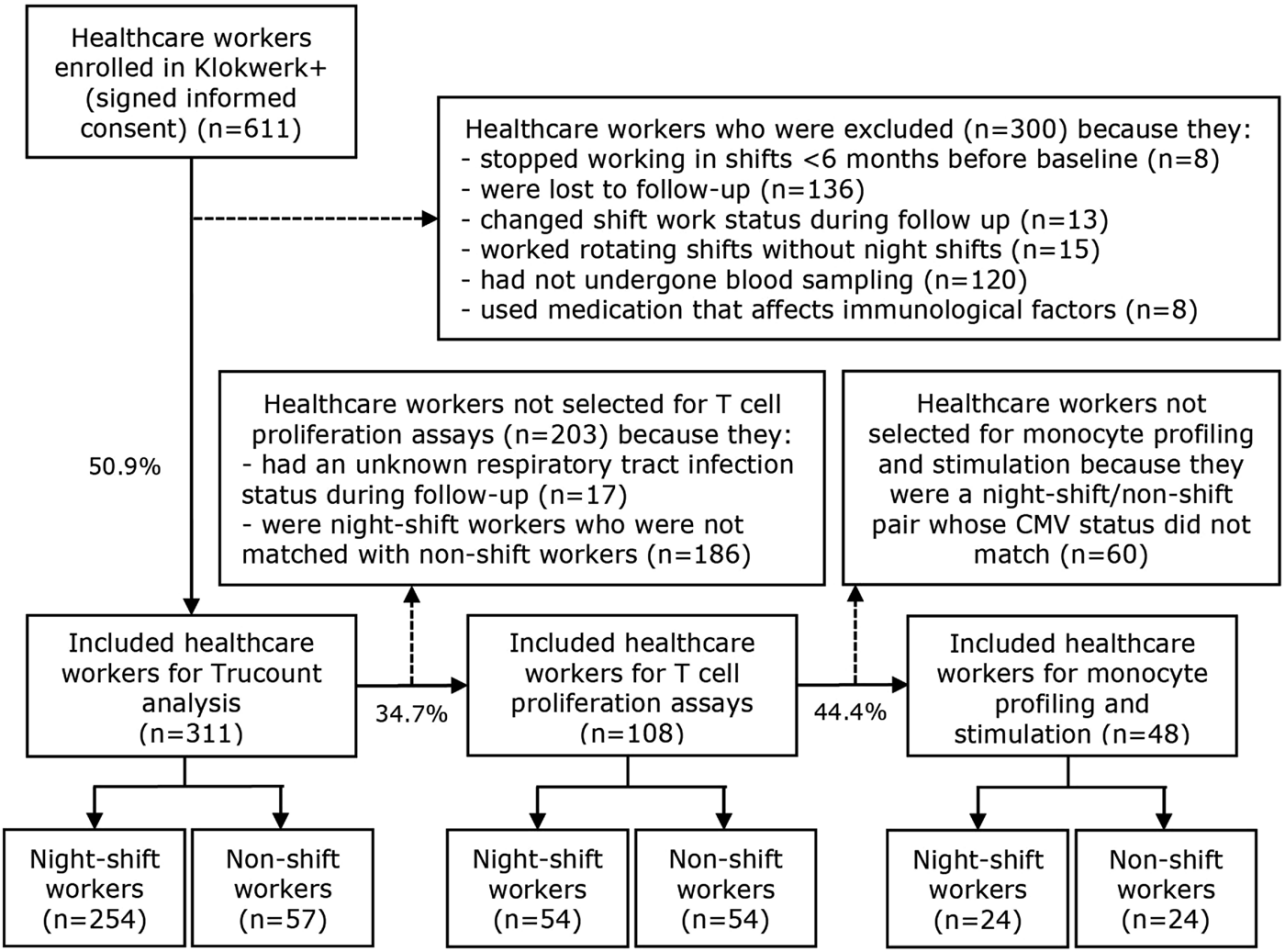
Flowchart of study participants.

Of the approximately 18,000 healthcare workers who were approached to participate in Klokwerk+, 1,227 filled in the online enrolment form. After exclusion of those who did not respond or declined to participate, 611 eligible workers enrolled in Klokwerk+. Of these, 254 night-shift and 57 non-shift workers were included for the Trucount analysis (Fig. 1). As shown in Table 1, night-shift workers were on average younger than non-shift workers (42.1 years (SD = 11.9) vs. 47.4 (SD = 9.9)), more often nurses (83.1% vs. 35.1%), and less often higher educated (53.9% vs.71.9%) (p < 0.05). Similar differences in demographics between night- and non-shift workers were found in the total study population of Klokwerk+^6^. For the T cell proliferation assays, 54 night-shift and 54 non-shift workers who were matched on age and gender were selected (Fig. 1). As not every participant had enough PBMC available (<1 * 10^6^ per stimulus) to perform proliferation assays using all six stimulus types, the following number of night-shift and non-shift worker pairs were available per stimulus: αCD3αCD28 52 pairs, TT 54 pairs, sCEFX 49 pairs, PHA 38 pairs, SEB 32 pairs, and sEFX 23 pairs. For the monocyte profiling and stimulation assays, 24 night-shift workers and 24 non-shift workers who were matched on age, gender, and CMV status were selected (Fig. 1).

**Table 1:** Characteristics of the study population stratified for night-shift and non-shift workers.

	Night-shift workers (n = 254) *% (n)*	Non-shift workers (n = 57) *% (n)*
Age (in years, mean (SD))	42.1* (11.9)	47.4* (9.9)
Gender (% female)	88.6 (225)	86.0 (49)
CMV status (% positive)	38.2 (97)	38.6 (22)
Occupation (% nurse)	83.1* (211)	35.1* (20)
Educational level (% high)	53.9* (137)	71.9* (41)
Marital status (% married/living together)	76.0 (193)	77.2 (44)
General health (% very good/excellent)	50.4 (128)	54.4 (31)
Smoker (% yes)	9.4 (24)	5.3 (3)
Recent infection (% yes)	11.0 (28)	10.5 (6)
Influenza vaccination (% yes)	14.6 (37)	24.6 (14)
Chronotype (%)		
Morning type	34.3* (87)	57.9* (33)
Evening type	42.1* (107)	22.8* (13)
Intermediate type	23.6 (60)	19.3 (11)
Frequency of night shifts (%)		—
1–2 night shifts/month	17.3 (44)	
3–4 night shifts/month	47.2 (120)	
≥5 night shifts/month	35.4 (90)	
Years of night-shift work (%)		—
<10 years	33.1 (84)	
10–19 years	24.4 (62)	
≥20 years	42.5 (108)	
Night shift in past three days (% yes)	22.8 (58)	—

*Statistically significant difference (p < 0.05) between night-shift workers and non-shift workers tested using the independent-samples t-test and chi-square test.

### Number of monocytes in night-shift and non-shift workers

To assess differences in immune cell numbers between night-shift and non-shift workers, absolute cell counts of monocytes, granulocytes, lymphocytes, and T cell subsets were determined (Table 2). Night-shift workers had a significantly higher number of monocytes (median (interquartile range): 403.1 (171.3) vs. 362.5 (184.0) cells/μl blood) in their blood than non-shift workers. After adjustment for covariates, the difference in number of monocytes remained statistically significant, with the geometric mean of night-shift workers being 1.15 (95%-CI = 1.05–1.26) times as large as that of non-shift workers (Table 2). The higher number of monocytes among night-shift workers was irrespective of recent night-shift work, frequency of night shifts, duration of night-shift work, and chronotype of night-shift workers (Table 3 and Supplementary Tables S1 and 2).

**Table 2:** Differences in immune cell counts in blood between night-shift workers and non-shift workers.

Cells/μl blood	Night-shift workers (n = 254)		Non-shift workers (n = 57)		Night-shift workers vs. non-shift workers
	*mean (SD)*	*median (IQR)*	*mean (SD)*	*median (IQR)*	*B (95%-CI)*^*a,b*^
Monocytes	423.9* (133.7)	403.1* (171.3)	378.1* (135.5)	362.5* (184.0)	1.15* (1.05–1.26)
Granulocytes	3958.5 (1515.0)	3723.0 (1603.3)	3751.3 (1329.0)	3570.5 (1493.4)	1.04 (0.93–1.16)
Lymphocytes	2109.3 (660.2)	2019.4 (775.0)	1953.3 (557.3)	1925.4 (734.9)	1.04 (0.95–1.14)
NK cells	236.1 (124.7)	212.7 (156.6)	263.2 (117.1)	238.2 (137.2)	0.89 (0.76–1.05)
NKT cells	83.0 (83.6)	57.1 (72.4)	76.1 (66.4)	52.9 (81.6)	1.03 (0.81–1.31)
B cells	243.6 (126.2)	219.1 (137.8)	217.4 (99.5)	210.2 (131.3)	1.05 (0.91–1.22)
T cells	1545.8* (523.8)	1479.7* (628.4)	1397.3* (462.0)	1328.9* (675.1)	1.05 (0.96–1.16)
CD4 T cells	976.1 (348.4)	936.0 (433.6)	927.6 (333.6)	871.2 (361.3)	1.02 (0.92–1.13)
CD4 effector memory T cells	105.8 (63.5)	90.7 (64.4)	103.2 (62.2)	85.3 (60.9)	1.08 (0.94–1.24)
CD4 central memory T cells	359.7 (142.8)	334.4 (163.7)	339.4 (130.4)	318.4 (179.4)	1.03 (0.93–1.15)
CD4 naive T cells	489.5 (243.9)	457.5 (305.2)	461.8 (242.0)	397.0 (227.1)	0.97 (0.83–1.14)
CD4 effector T cells	21.1 (29.9)	9.6 (18.2)	23.2 (35.5)	11.1 (19.5)	0.84 (0.63–1.13)
CD8 T cells	443.8* (213.9)	417.7* (249.4)	360.3* (193.0)	317.6* (229.0)	1.14 (1.00–1.29)
CD8 effector memory T cells	32.8 (30.2)	25.8 (25.7)	31.7 (35.4)	19.1 (18.7)	1.20 (0.96–1.50)
CD8 central memory T cells	77.7* (43.2)	65.9* (52.9)	64.8* (35.8)	54.6* (53.5)	1.17 (0.99–1.38)
CD8 naive T cells	225.6* (129.8)	194.9* (159.0)	168.6* (84.3)	149.2* (133.5)	1.08 (0.92–1.26)
CD8 effector T cells	107.7 (119.9)	68.0 (98.9)	95.3 (98.2)	53.2 (102.5)	1.01 (0.81–1.27)
CD4/CD8 T cell ratio	2.6* (1.6)	2.2* (1.4)	3.1* (1.6)	2.9* (1.7)	0.90 (0.80–1.02)

B, regression coefficient; CI, confidence interval; IQR, interquartile range; SD, standard deviation.

T cell subsets: Effector memory T cells: CD27−/CD45RO+, Central memory T cells: CD27+/CD45RO+, Naive T cells: CD27+/CD45RO−, Effector T cells: CD27−/CD45RO−.

*Statistically significant difference (p < 0.05) between night-shift workers and non-shift workers tested using the independent-samples t-test for normally distributed outcomes (column 1 and 2), the Mann-Whitney U test for non-normally distributed outcomes (column 1 and 2), and adjusted mixed model regression analysis for the log-transformed outcomes (column 3).

^a^The regression coefficients are ratios between geometric means of night-shift workers and non-shift workers.

^b^Adjusted for age, gender, CMV status, occupation, educational level, general perceived health, smoking status, influenza vaccination status, and recent infection.

**Table 3:** Effect estimates of the differences in immune cell counts in blood by recent night-shift work (past three days) compared to non-shift workers.

Cells/μl blood	Night-shift workers with no night shift in past three days (n = 196) *B (95%-CI)*^*a,b*^	Night-shift workers with night shift in past three days (n = 57) *B (95%-CI)*^*a,b*^
Monocytes	1.15* (1.05–1.27)	1.14* (1.01–1.28)
Granulocytes	1.05 (0.94–1.18)	0.97 (0.84–1.12)
Lymphocytes	1.03 (0.94–1.12)	1.12*^c^ (1.01–1.26)
NK cells	0.90 (0.76–1.05)	0.89 (0.72–1.08)
NKT cells	1.02 (0.80–1.30)	1.10 (0.82–1.48)
B cells	1.03 (0.89–1.20)	1.16 (0.96–1.39)
T cells	1.04 (0.94–1.14)	1.16*^c^ (1.03–1.31)
CD4 T cells	1.00 (0.90–1.11)	1.13^c^ (0.99–1.29)
CD4 effector memory T cells	1.07 (0.93–1.23)	1.12 (0.94–1.34)
CD4 central memory T cells	1.02 (0.91–1.13)	1.13^c^ (0.99–1.30)
CD4 naive T cells	0.94 (0.80–1.11)	1.13^c^ (0.92–1.38)
CD4 effector T cells	0.83 (0.62–1.12)	0.89 (0.61–1.29)
CD8 T cells	1.12 (0.98–1.27)	1.23* (1.05–1.45)
CD8 effector memory T cells	1.19 (0.95–1.49)	1.24 (0.94–1.65)
CD8 central memory T cells	1.14 (0.97–1.35)	1.30* (1.05–1.61)
CD8 naive T cells	1.05 (0.89–1.23)	1.25*^c^ (1.02–1.52)
CD8 effector T cells	1.02 (0.81–1.29)	0.96 (0.72–1.29)
CD4/CD8 T cell ratio	0.90 (0.79–1.01)	0.92 (0.79–1.07)

Reference group: non-shift workers (n = 57).

B, regression coefficient; CI, confidence interval.

T cell subsets: Effector memory T cells: CD27−/CD45RO+, Central memory T cells: CD27+/CD45RO+, Naive T cells: CD27+/CD45RO−, Effector T cells: CD27−/CD45RO−.

*Statistically significant difference (p < 0.05) between night-shift workers and non-shift workers tested using linear regression analysis for the log-transformed outcomes.

^a^The regression coefficients are ratios between geometric means of night-shift workers and non-shift workers.

^b^Adjusted for age, gender, CMV status, occupation, educational level, general perceived health, smoking status, influenza vaccination status, and recent infection.

^c^Statistically significant difference (p < 0.05) between night-shift workers with night shift in past three days and night-shift workers with no night shift in past three days tested using linear regression analysis for the log-transformed outcomes.

### Functionality of monocytes in night-shift and non-shift workers

As an altered composition of monocyte subsets could be indicative of a more proinflammatory state in night-shift workers, the phenotype of the monocytes in a subsample of the study population (n = 48, Fig. 1) was also assessed. In this subsample, a similar effect size for the increased monocyte count among night-shift workers was observed (B = 1.14 (95%-CI = 0.91–1.44)). Analysis of the profile of the monocytes showed that percentages of classical (CD14high/CD16low), intermediate (CD14high/CD16intermediate), and non-classical (CD14intermediate/CD16high) monocytes were similar in night-shift and non-shift workers (Supplementary Fig. S4). In addition, no evidence was found for different cytokine profiles after stimulation of PBMC with TLR ligands (the cytokine profile of IL-6 is shown as example in Supplementary Fig. S5). Furthermore, no differences between night-shift and non-shift workers were found in the same cytokines and chemokines in serum, except for a higher concentration of chemokine Macrophage Inflammatory Protein 1β (MIP-1β/CCL4) (B = 1.39 (95%-CI = 1.10–1.77)) in night-shift workers. In addition, night-shift workers who worked night shifts recently also had a higher concentration of cytokine Interleukin-18 (IL-18) (B = 1.98 (95%-CI = 1.27–3.08)) than non-shift workers.

### Number of lymphocytes in night-shift and non-shift workers

Night-shift workers had a higher number of T cells (median = 1479.7 (IQR = 628.4) vs. median = 1328.9 (IQR = 675.1) cells/μl blood), and especially CD8 T cells (median = 417.7 (IQR = 249.4) vs. median = 317.6 (IQR = 229.0) cells/μl blood) than non-shift workers (Table 2). These differences seemed to be due to the shift workers who worked night shifts in the past three days. Even after adjustment for covariates, they had significantly higher numbers of lymphocytes (B = 1.12 (95%-CI = 1.01–1.26)), T cells (B = 1.16 (95%-CI = 1.03–1.31)), and CD8 T cells (B = 1.23 (95%-CI = 1.05–1.45)) than non-shift workers (Table 3). As known parameters to be associated with impaired immune function are a higher number of effector T cells, a lower number of naive T cells, and a lower CD4/CD8 ratio^30^, differences in T cell subsets were also examined. Table 2 shows that after adjustment for covariates, there were no statistically significant differences in CD4 and CD8 T cell subsets between night-shift and non-shift workers. Although night-shift workers had a lower CD4/CD8 ratio than non-shift workers in the crude analysis, this difference did not remain statistically significant after adjustment for covariates. No trend in numbers of immune cells was observed for frequency and duration of night-shift work (Supplementary Table S1).

### T cell proliferative responses in night-shift and non-shift workers

**Figure 2:**
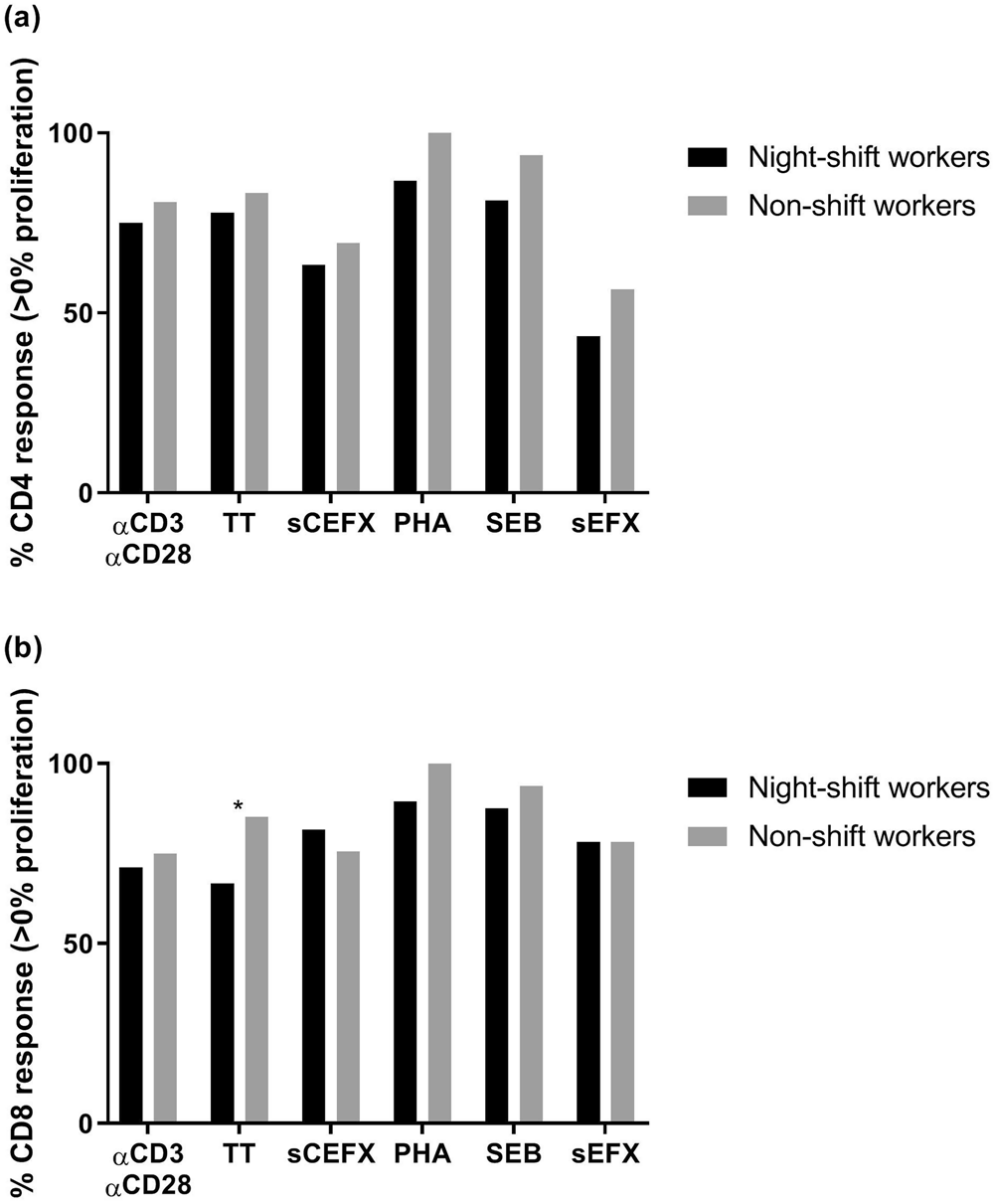
Proportions of night-shift workers (black) and non-shift workers (grey) who expressed CD4 T cell (**a**) and CD8 T cell (**b**) proliferation as a response to the different stimuli (>0% stimulus-specific T cell proliferation). An asterisk indicates a statistically significant difference (p < 0.05) between night-shift and non-shift workers tested using the chi-square test.

**Figure 3:**
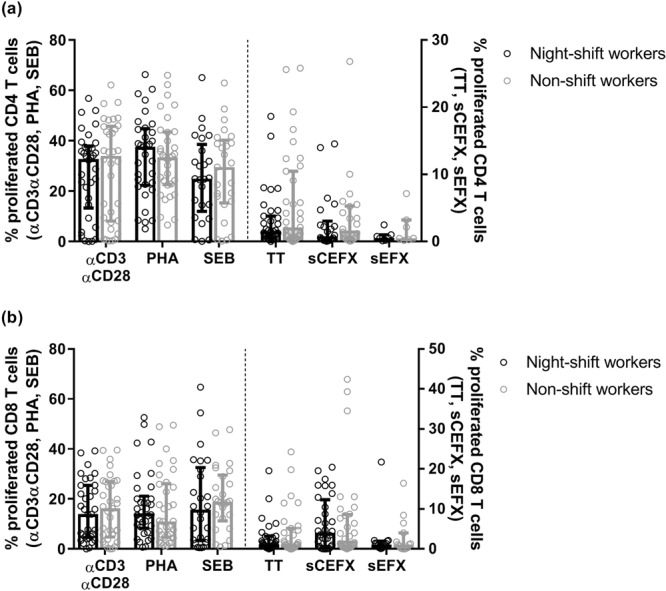
Percentages proliferated CD4 T cells (**a**) and CD8 T cells (**b**) in night-shift workers (black) and non-shift workers (grey) among the responders (>0% stimulus-specific T cell proliferation). Bars indicate median with interquartile range.

Next, we assessed whether night-shift work may affect immune function of lymphocytes by determining the proliferative capacity of T cells after stimulation with different T cell stimuli (i.e. αCD3αCD28, TT, sCEFX, PHA, SEB, and sEFX). By identifying the percentage of cells with celltrace dye dilution, the percentages of divided precursors were determined per stimulus (Supplementary Fig. S3). Overall, the proportion of participants who expressed stimulus-specific T cell proliferation (>0%) was highest for stimuli PHA and SEB (Supplementary Fig. S6). For both CD4 and CD8 T cells, between 90% and 96% of participants responded to these stimuli. Correspondingly, among the responders (>0% proliferation), the percentage of proliferated T cells was highest for stimuli PHA, SEB, and αCD3αCD28 (Supplementary Fig. S7). The percentage of proliferated T cells after stimulation with TT, sCEFX, and sEFX was generally low, with a median percentage response below 3%. Due to the low overall response to these stimuli, it may be difficult to determine clear differences between night-shift and non-shift workers. The proportions of night-shift workers who expressed stimulus-specific CD4 and CD8 T cell proliferation (>0%) were generally lower than the proportions of non-shift workers, although a statistically significant difference was only found for TT, to which 68% of night-shift workers and 85% of non-shift workers expressed CD8 T cell proliferation (p < 0.05) (Fig. 2). Among the responders (>0% proliferation), percentages of CD4 and CD8 T cell proliferation (Fig. 3) and odds ratios of high stimulus-specific T cell proliferation (Table 4) were similar for night-shift and non-shift workers. For all stimuli combined, night-shift workers did not have significantly lower odds of expressing CD4 (OR = 0.56 (95%-CI = 0.28–1.11)) and CD8 (OR = 0.53 (95%-CI = 0.27–1.03)) T cell proliferation, and of having high CD4 (OR = 1.38 (95%-CI = 0.76–2.51)) and CD8 (OR = 0.93 (95%-CI = 0.57–1.51)) T cell proliferation among the responders (Table 4). In addition, no differences between night-shift and non-shift workers were found in concentration of the cytokines measured in supernatant of PMBC stimulated with T cell stimuli (Supplementary Table S3).

**Table 4:** Odds ratios of expressing CD4 and CD8 T cell proliferation (i.e. response vs. no response, based on >0% proliferation vs. 0% proliferation) and odds ratios of high (i.e. upper 50% of observations) CD4 and CD8 T cell proliferation among the responders, compared between night-shift workers (n = 54) and non-shift workers (ref, n = 54).

T cells	Stimulus	Night-shift workers vs. non-shift workers			
		Response vs. no response^a^		High vs. low response among responders^b^	
		Per stimulus *OR (95%-CI)*^c^	Combined *OR (95%-CI)*^c,d^	Per stimulus *OR (95%-CI)*^c^	Combined *OR (95%-CI)*^c,d^
CD4	αCD3αCD28	0.51 (0.17–1.58)	0.56 (0.28–1.11)	1.19 (0.37–3.78)	1.38 (0.76–2.51)
	TT	0.65 (0.20–2.09)		0.52 (0.16–1.68)	
	sCEFX	0.56 (0.20–1.54)		1.36 (0.31–6.05)	
	PHA	NA		2.94 (0.83–10.36)	
	SEB	0.30 (0.04–2.25)		4.17 (0.70–24.80)	
	sEFX	0.55 (0.13–2.34)		1.20 (0.05–29.37)	
CD8	αCD3αCD28	0.65 (0.23–1.85)	0.53 (0.27–1.03)	0.59 (0.17–1.99)	0.93 (0.57–1.51)
	TT	0.22 (0.06–0.76)*		0.51 (0.14–1.82)	
	sCEFX	0.93 (0.30–2.95)		2.23 (0.68–7.29)	
	PHA	NA		1.37 (0.45–4.21)	
	SEB	0.38 (0.04–3.27)		0.84 (0.19–3.64)	
	sEFX	0.60 (0.10–3.67)		0.42 (0.06–3.09)	

CI, confidence interval; OR, odds ratio.

NA: Analysis not possible due to empty cell (0 non-shift workers were non-responders to PHA).

^a^The following number of pairs of night-shift and non-shift workers were available for analyses per stimulus type: αCD3αCD28 n = 52, TT n = 54, sCEFX n = 49, PHA n = 38, SEB n = 32, and sEFX n = 23.

^b^The following number of pairs of night-shift and non-shift workers were available for analyses per stimulus type for CD4/CD8: αCD3αCD28 n = 34/33, TT n = 35/31, sCEFX n = 22/32, PHA n = 33/34, SEB n = 25/26, and sEFX n = 7/15.

^c^Adjusted for CMV status and occupation.

^d^Odds ratios combined for all stimuli, using logistic Generalized Estimating Equations (GEE) analysis.

*p < 0.05 (tested using logistic regression analysis).

## Discussion

In this study among healthcare workers, differences between night-shift and non-shift workers in number as well as function of immune cells were studied. Night-shift workers had a higher number of monocytes than non-shift workers. Furthermore, night-shift workers who worked night shifts in the past three days had significantly more lymphocytes, T cells, and CD8 T cells than non-shift workers. With respect to function of immune cells, the results indicated no large differences in monocyte functionality and T cell proliferative responses to various stimuli between night-shift and non-shift workers.

One of the main results of the current study is the elevated level of monocytes in night-shift workers compared to non-shift workers. The higher number of monocytes was prevalent in all night-shift workers, regardless of night-shift work frequency, duration, and recency of night-shift work. This suggests that night-shift work might have a robust, long-lasting influence on these innate immune cells. An explanation for this finding might be that circadian rhythm disruption induced by night-shift work disturbs the function of clock proteins (e.g. BMAL1) that normally attenuate monocyte numbers^31^. Besides the fact that monocytes play an important role in the defence mechanism against infection, an increased level of monocytes has been found to be associated with health consequences such as atherosclerosis, tumour metastasis, and ageing and frailty^32–34^. Furthermore, the absence of certain clock proteins in mice was shown to lead to an increase in monocyte numbers^35^, and is proposed to be associated with metabolic diseases and obesity^31,35,36^. Due to the relation between monocytes and health^31–35^, it can be concluded that the observed elevation in monocytes may play a mechanistic role in the negative health effects of shift work, such as the increased risk for cardiovascular and infectious diseases. Future research should be undertaken to explore this potential mechanistic role.

Alterations in proinflammatory cytokines such as TNF-α, IL-6, and IL-1β could be expected among night-shift workers, as these markers are secreted in a circadian manner^37^ and are associated with sleep disturbances^38,39^. However, in the current study as well as previous studies, no obvious alterations in these proinflammatory cytokines were observed when comparing shift workers with non-shift workers^11,23^. In the current study, night-shift workers did have a higher concentration of MIP-1β and, after working night shifts recently, IL-18. MIP-1β and IL-18 are both produced by monocytes/macrophages^40,41^. As MIP-1β is a chemokine that attracts monocytes^40^, this finding may be related to the observed elevated level of monocytes in night-shift workers. Correspondingly, additional analysis revealed that a higher concentration of MIP-1β was significantly associated with a higher number of monocytes. However, no associations between night-shift work and the wide range of other cytokines and chemokines were found (such as the innate cytokine IL-6 and the more potent monocyte-attracting monocyte MIP-1α/CCL3^40^), and therefore these findings should be interpreted with caution.

While the observed higher number of monocytes among night-shift workers was irrespective of recent night-shift work, for lymphocytes, the results of this study indicate that recent night-shift work may have a larger effect on the number of lymphocytes, T cells, and CD8 T cells than general night-shift work status. Similarly, Wirth *et al*. (2017) reported larger differences in number of lymphocytes when comparing short-term night-shift work to day work, than when comparing long-term night-shift work status to day work^21^. Thus, after working night shifts, counts of these immune cells may be disturbed, but this change may restore when the night-shift worker has stopped working night shifts for some time, and therefore demonstrate an acute effect of night-shift work and not a long-term effect. As many aspects of the adaptive immune response and T cell functions vary across the day^42^, it is also possible that the reported differences in the recent night-shift work group simply reflect the fact that this group was in a different stage of the circadian rhythm of immune cells at the time of blood sample collection^43^. However, Cuesta *et al*. (2016) reported that the rhythm in monocytes and T cells was not shifted after working simulated night shifts, which suggests that a rhythm shift in these parameters might not explain our findings^10^. Recent studies examining clock gene expression and resetting in night-shift work have indicated that peripheral clock gene expression in PBMC is disrupted by working night shifts under real-life working conditions^44^ and that light exposure at night has a large impact on the resetting of peripheral circadian clocks^45^. Furthermore, large variability in the degree of misalignment in circadian gene expression rhythms among individual night-shift workers has been reported^46^. As alterations in these expression profiles in PBMC may be related to subsequent changes in numbers and function of immune cells, studying immunological disturbances in night-shift workers would benefit from taking into account the complete rhythm of immune cells during the day and night. Because we collected blood samples only at one time point, a limitation of the current study is that it was not possible to determine the 24 hour rhythm of the PBMC. Therefore, for future research, it is recommended to collect multiple blood samples to examine if the reported differences are due to an elevation of immune cells directly after night shifts or due to recent night-shift workers being in a different stage of the circadian rhythm of immune cells. Furthermore, in order to gain more insight into the extent of the effect of night-shift work on the immune system, it would be interesting to study how long it takes for disturbances in (the circadian rhythm of) immune cells to restore after working night shifts.

With respect to the association between night-shift work and cellular immune functions, we observed no large differences in T cell proliferative responses to various stimuli. Similarly, Copertaro *et al*. (2011) observed no differences in lymphocyte proliferative response to PHA between shift working nurses and daytime nurses, and concluded that the immune response to shift work is probably highly variable and subject to changes over time^23^. In accordance with this conclusion, Nakano *et al*. (1982) reported that the T cell function of shift workers might be differently affected according to the pattern of shift work^24^. For example, they found that rotating shift workers’ PHA response of T cells was lower than the response of non-shift workers when blood samples were collected in the evening, but responses were similar when blood samples were collected in the morning^24^. Therefore, it is possible that the results of the current study were diminished by that the effect of recent night-shift work was not taken into account in the selection of night-shift workers for the T cell proliferation assays and by that the blood sample collection were collected only in the morning.

Based on the current and previous results, it is likely that some immune cells and functions are affected by shift work, while others are not, thereby losing the close synchronization between these cells and functions^10^. For example, CD8 T cells were elevated after recent night-shift work, while numbers of CD4 T cells did not differ, leading to a lower CD4/CD8 ratio, which has been found to be associated with negative health outcomes^47^. Previous research also indicates that circadian rhythm disruption leads to increased virus replication and dissemination^48^, potentially making shift workers more vulnerable to infection when their circadian rhythm of immune cells is disturbed. In addition, as responses to pathogens differ during the day^37,48,49^, with probably being most susceptible to infection during the normal rest phase, shift workers starting a night shift may already be more prone to infection. As night-shift work may affect the immune status, shift work schedule should be taken into account in strategies for the prevention of negative health effects in shift workers, such as timing of vaccination. For example, vaccination in shift workers may be less effective after recent night-shift work^9^, and further research on optimal vaccination strategies in shift workers is therefore needed.

In conclusion, in this study among healthcare workers, night-shift workers had higher levels of monocytes than non-shift workers. Furthermore, numbers of lymphocytes, T cells, and CD8 T cells were higher in shift workers who worked night shifts in the past three days. Despite these differences, no large differences were found in numbers of other immune cells and in functional aspects of monocytes and T cells as exemplified by monocyte cytokine production and T cell proliferative responses to various stimuli. Nonetheless, the results of this study suggest that chronic exposure to night-shift work as well as recent night-shift work may influence the immune status of workers, which could be relevant knowledge for preventive initiatives in night-shift workers.

## Supplementary information

Supplementary Figures and Tables

## Data Availability

The datasets generated during and analysed during the current study are available from the corresponding author on reasonable request.
