# Supplementary material for: Immunological effects of shift work in healthcare workers
Source: Sci Rep. 2019 Dec 3;9:18220. doi: 10.1038/s41598-019-54816-5 (PMC6890754; doi:10.1038/s41598-019-54816-5)
Supplement: Supplementary file 1 — Supplementary Figures and Tables [file 41598_2019_54816_MOESM1_ESM.pdf]

Supplementary information

Immunological effects of shift work in healthcare workers

Bette Loef, Nening M Nanlohy, Ronald H J Jacobi, Chantal van de Ven, Rob Mariman, Allard J van der Beek, Karin I Proper, and Debbie van Baarle

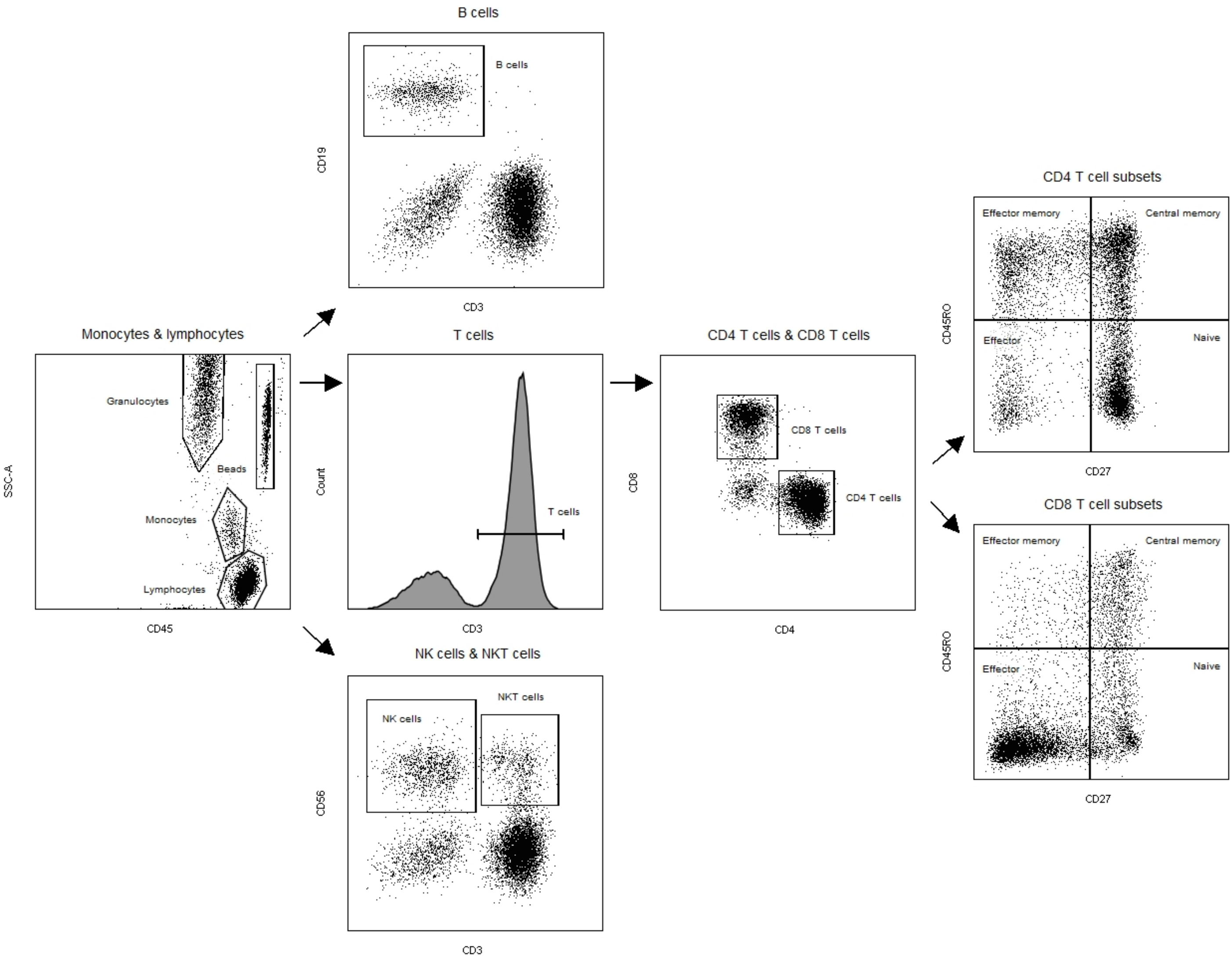

Supplementary Figure S1. Gating strategy used to determine numbers of immune cells.

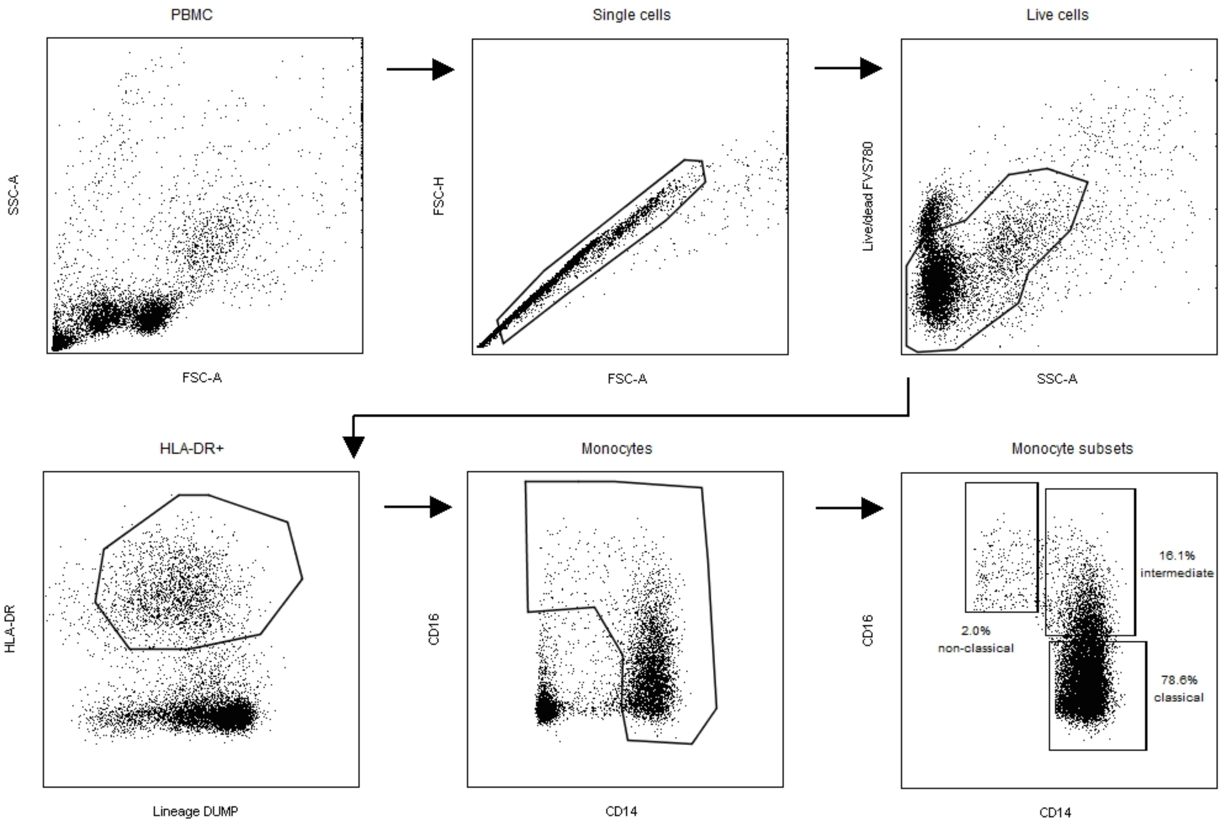

Supplementary Figure S2. Gating strategy used to determine monocyte subsets.

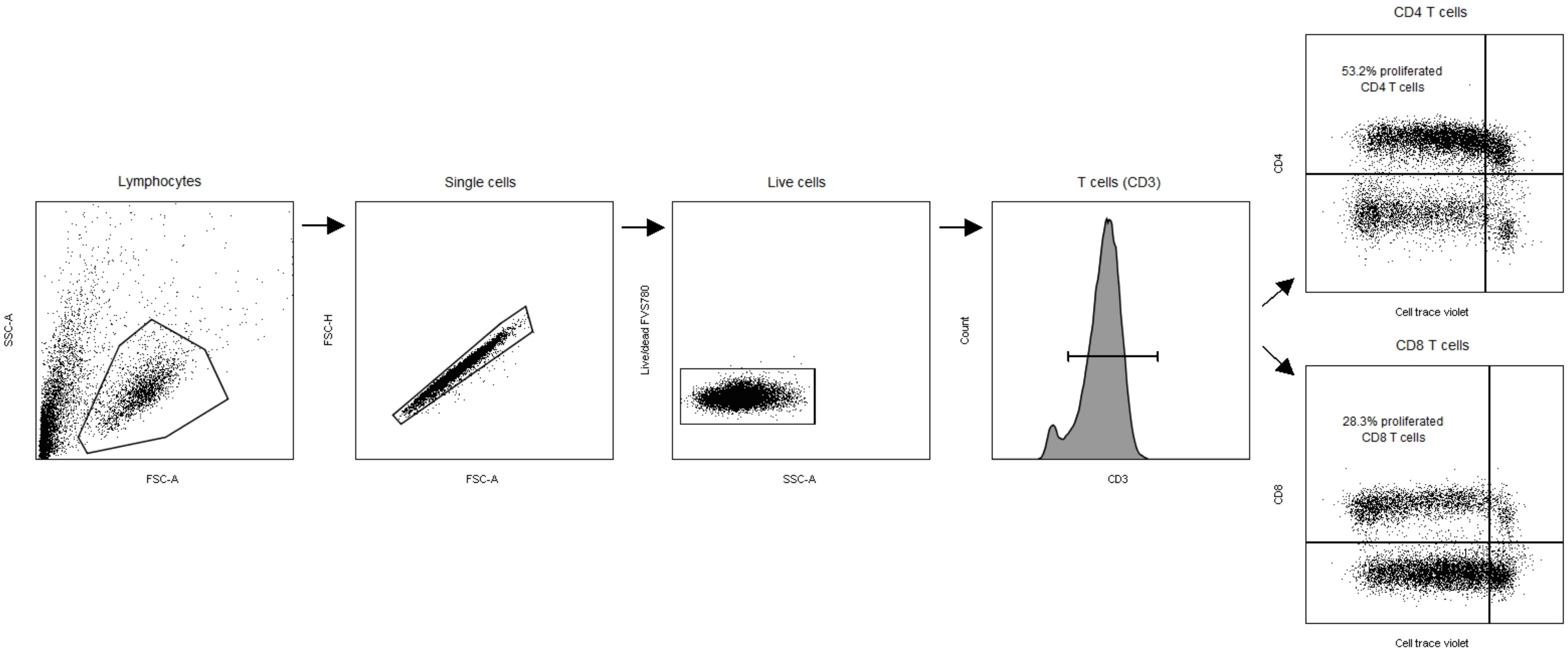

Supplementary Figure S3. Gating strategy used to determine CD4 and CD8 T cell proliferation. The example depicts the proliferative response of a participant to αCD3αCD28.

Supplementary Table S1. Effect estimates of the differences in immune cell counts in blood by frequency of night shifts and duration of night-shift work, compared to non-shift workers

	Frequency of night shifts			Duration of night-shift work		
Cells/µl blood	1–2 night shifts/month (n=44) *B (95%-CI)*[a,b]	3–4 night shifts/month (n=120) *B (95%-CI)*[a,b]	≥5 night shifts/month (n=90) *B (95%-CI)*[a,b]	<10 years (n=84) *B (95%-CI)*[a,b]	10–19 years (n=62) *B (95%-CI)*[a,b]	≥20 years (n=108) *B (95%-CI)*[a,b]
Monocytes	1.17* (1.04 – 1.32)	1.14* (1.03 – 1.26)	1.15* (1.03 – 1.28)	1.15* (1.01 – 1.30)	1.16* (1.03 – 1.29)	1.15* (1.04 – 1.28)
Granulocytes	1.04 (0.90 – 1.19)	1.06 (0.93 – 1.19)	1.02 (0.90 – 1.16)	1.05 (0.90 – 1.21)	1.04 (0.91 – 1.20)	1.03 (0.91 – 1.17)
Lymphocytes	1.03 (0.93 – 1.16)	1.02 (0.92 – 1.12)	1.08 (0.97 – 1.19)	1.08 (0.96 – 1.22)	1.07 (0.96 – 1.19)	1.01 (0.91 – 1.11)
NK cells	0.84 (0.69 – 1.02)	0.92 (0.77 – 1.09)	0.92 (0.77 – 1.11)	0.84 (0.68 – 1.04)	0.93 (0.77 – 1.13)	0.90 (0.76 – 1.08)
NKT cells	1.06 (0.79 – 1.43)	1.06 (0.82 – 1.38)	0.97 (0.74 – 1.28)	1.09 (0.80 – 1.50)	1.09 (0.82 – 1.45)	0.98 (0.75 – 1.28)
B cells	1.06 (0.89 – 1.28)	1.01 (0.86 – 1.18)	1.10 (0.93 – 1.30)	1.08 (0.89 – 1.32)	1.08 (0.90 – 1.29)	1.02 (0.87 – 1.21)
T cells	1.06 (0.94 – 1.20)	1.03 (0.92 – 1.14)	1.09 (0.97 – 1.22)	1.10 (0.97 – 1.25)	1.09 (0.97 – 1.23)	1.02 (0.91 – 1.13)
CD4 T cells	1.03 (0.91 – 1.18)	0.99 (0.88 – 1.11)	1.03 (0.92 – 1.17)	1.06 (0.92 – 1.22)	1.04 (0.92 – 1.19)	0.98 (0.87 – 1.10)
CD4 effector memory T cells	1.18 (0.99 – 1.413)	1.00 (0.86 – 1.17)	1.08 (0.92 – 1.27)	1.12 (0.93 – 1.36)	1.14 (0.96 – 1.35)	1.03 (0.88 – 1.20)
CD4 central memory T cells	1.10 (0.96 – 1.26)	1.00 (0.88 – 1.12)	1.03 (0.91 – 1.16)	1.02 (0.88 – 1.18)	1.11 (0.98 – 1.27)	1.01 (0.89 – 1.14)
CD4 naive T cells	0.92 (0.75 – 1.12)	0.98 (0.82 – 1.17)	1.01 (0.84 – 1.22)	1.08 (0.87 – 1.34)	0.95 (0.78 – 1.15)	0.93 (0.78 – 1.12)
CD4 effector T cells	0.93 (0.64 – 1.34)	0.76 (0.55 – 1.06)	0.88 (0.63 – 1.24)	1.14 (0.77 – 1.70)	0.95 (0.67 – 1.36)	0.69* (0.50 – 0.96)
CD8 T cells	1.14 (0.97 – 1.34)	1.09 (0.94 – 1.25)	1.20* (1.04 – 1.40)	1.19 (1.00 – 1.41)	1.21* (1.04 – 1.42)	1.08 (0.93 – 1.25)
CD8 effector memory T cells	1.26 (0.95 – 1.67)	1.11 (0.87 – 1.42)	1.25 (0.97 – 1.62)	1.16 (0.86 – 1.57)	1.34* (1.02 – 1.75)	1.16 (0.90 – 1.49)
CD8 central memory T cells	1.31* (1.07 – 1.62)	1.11 (0.92 – 1.33)	1.12 (0.92 – 1.36)	1.13 (0.90 – 1.42)	1.32* (1.08 – 1.62)	1.12 (0.93 – 1.35)
CD8 naive T cells	1.03 (0.85 – 1.26)	1.01 (0.85 – 1.20)	1.21* (1.01 – 1.45)	1.18 (0.95 – 1.46)	1.11 (0.91 – 1.34)	1.02 (0.85 – 1.22)
CD8 effector T cells	1.15 (0.86 – 1.54)	0.94 (0.73 – 1.21)	0.99 (0.76 – 1.29)	1.00 (0.73 – 1.36)	1.12 (0.84 – 1.48)	0.98 (0.75 – 1.26)
CD4/CD8 T cell ratio	0.91 (0.78 – 1.06)	0.92 (0.80 – 1.05)	0.86* (0.75 – 1.00)	0.90 (0.77 – 1.06)	0.87 (0.75 – 1.01)	0.92 (0.80 – 1.05)

Reference group: non-shift workers.

B, regression coefficient; CI, confidence interval. T cell subsets: Effector memory T cells: CD27-/CD45RO+, Central memory T cells: CD27+/CD45RO+, Naive T cells: CD27+/CD45RO-, Effector T cells: CD27-/CD45RO-.

* Statistically significant difference ($p<0.05$) between night-shift workers and non-shift workers tested using linear regression analysis for the log-transformed outcomes.

[a] The regression coefficients are ratios between geometric means of night-shift workers and non-shift workers.

[b] Adjusted for age, gender, CMV status, occupation, educational level, general perceived health, smoking status, influenza vaccination status, and recent infection.

Supplementary Table S2. Effect estimates of the differences in immune cell counts in blood by chronotype of night-shift workers, compared to non-shift workers

	Chronotype of night-shift workers		
Cells/µl blood	Night-shift workers with morning chronotype (n=87) *B (95%-CI)*[a,b]	Night-shift workers with evening chronotype (n=107) *B (95%-CI)*[a,b]	Night-shift workers with intermediate chronotype (n=60) *B (95%-CI)*[a,b]
Monocytes	1.16* (1.04 – 1.29)	1.14* (1.03 – 1.26)	1.16* (1.04 – 1.30)
Granulocytes	1.00 (0.89 – 1.14)	1.08 (0.95 – 1.22)	1.03 (0.90 – 1.18)
Lymphocytes	1.00 (0.91 – 1.11)	1.08 (0.98 – 1.19)	1.04 (0.93 – 1.15)
NK cells	0.83* (0.69 – 0.98)	0.92 (0.77 – 1.10)	0.96 (0.80 – 1.17)
NKT cells	0.98 (0.75 – 1.28)	1.07 (0.82 – 1.39)	1.06 (0.79 – 1.41)
B cells	0.99 (0.84 – 1.17)	1.13 (0.97 – 1.33)	1.01 (0.85 – 1.21)
T cells	1.02 (0.91 – 1.14)	1.09 (0.98 – 1.21)	1.05 (0.93 – 1.18)
CD4 T cells	0.98 (0.87 – 1.11)	1.06 (0.94 – 1.19)	0.99 (0.87 – 1.13)
CD4 effector memory T cells	1.10 (0.94 – 1.28)	1.06 (0.91 – 1.23)	1.08 (0.92 – 1.28)
CD4 central memory T cells	1.02 (0.91 – 1.15)	1.08 (0.96 – 1.22)	0.97 (0.86 – 1.11)
CD4 naive T cells	0.91 (0.76 – 1.09)	1.03 (0.87 – 1.23)	0.95 (0.78 – 1.15)
CD4 effector T cells	0.95 (0.68 – 1.32)	0.75 (0.54 – 1.03)	0.88 (0.62 – 1.26)
CD8 T cells	1.08 (0.93 – 1.25)	1.16* (1.01 – 1.34)	1.18* (1.01 – 1.38)
CD8 effector memory T cells	1.12 (0.87 – 1.44)	1.24 (0.97 – 1.59)	1.24 (0.95 – 1.63)
CD8 central memory T cells	1.08 (0.89 – 1.30)	1.23* (1.02 – 1.47)	1.20 (0.98 – 1.48)
CD8 naive T cells	1.02 (0.85 – 1.22)	1.12 (0.94 – 1.33)	1.09 (0.90 – 1.32)
CD8 effector T cells	1.02 (0.78 – 1.32)	0.99 (0.77 – 1.28)	1.06 (0.80 – 1.40)
CD4/CD8 T cell ratio	0.92 (0.80 – 1.05)	0.92 (0.80 – 1.05)	0.84* (0.73 – 0.97)

Reference group: non-shift workers.
B, regression coefficient; CI, confidence interval; IQR, interquartile range; SD, standard deviation.
T cell subsets: Effector memory T cells: CD27-/CD45RO+, Central memory T cells: CD27+/CD45RO+, Naive T cells: CD27+/CD45RO-, Effector T cells: CD27-/CD45RO-.
* Statistically significant difference ($p<0.05$) between night-shift workers and non-shift workers tested using linear regression analysis for the log-transformed outcomes.
[a] The regression coefficients are ratios between geometric means of night-shift workers and non-shift workers.
[b] Adjusted for age, gender, CMV status, occupation, educational level, general perceived health, smoking status, influenza vaccination status, and recent infection.

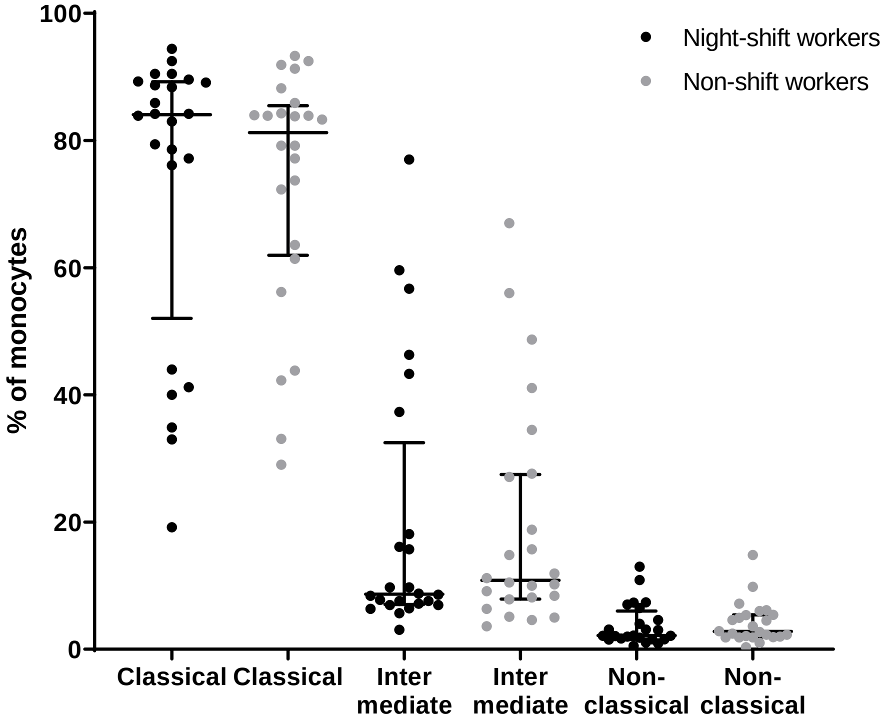

Supplementary Figure S4. Percentages of monocyte subsets (i.e. classical, intermediate, non-classical) in 24 night-shift workers (black) and 24 non-shift workers (grey) matched on age, gender, and CMV status.

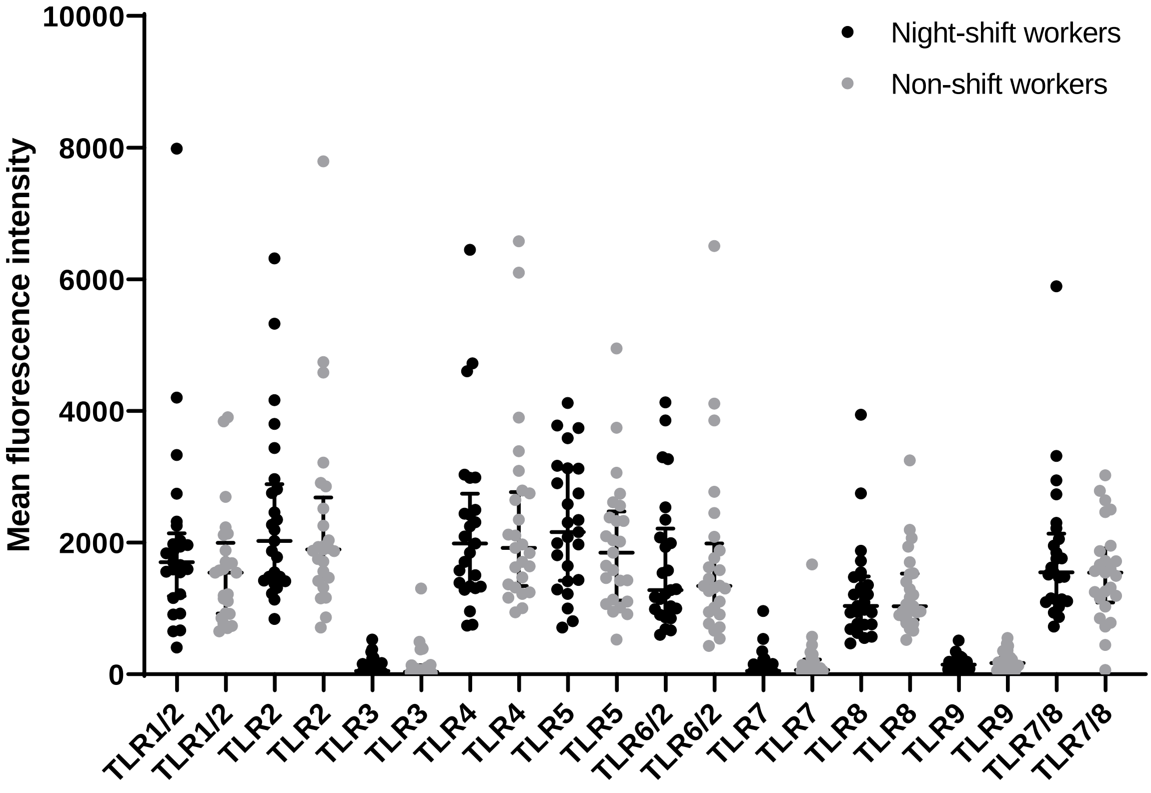

Supplementary Figure S5. Mean fluorescence intensity of IL-6 after stimulation of PBMC with TLR ligands in 24 night-shift workers (black) and 24 non-shift workers (grey) matched on age, gender, and CMV status.

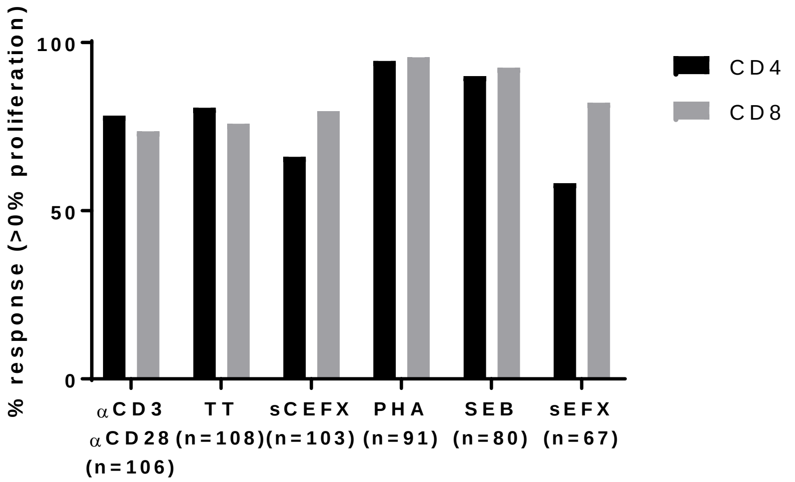

Supplementary Figure S6. Proportions of participants, irrespective of shift work status, who expressed CD4 T cell (black) and CD8 T cell (grey) proliferation as a response to the different stimuli (>0% stimulus-specific T cell proliferation).

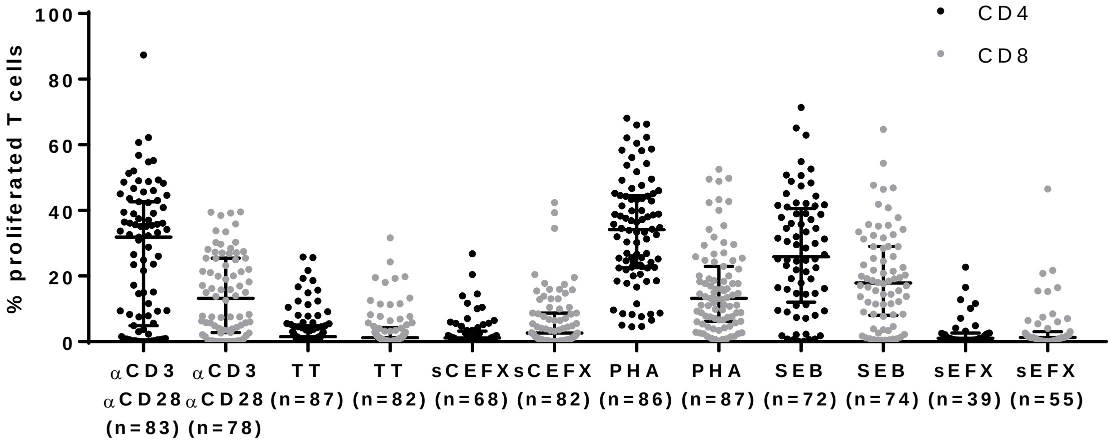

Supplementary Figure S7. Percentages proliferated CD4 T cells (black) and CD8 T cells (grey) among the responders (>0% stimulus-specific T cell proliferation), irrespective of shift work status.

Supplementary Table S3. Differences in concentration of cytokines measured in supernatant of PBMC stimulated with T-cell stimuli (after 1 and 6 days) between night-shift workers and non-shift workers

	Day 1		Day 6	
	Night shift workers (n=42)	Non-shift workers (n=42)	Night shift workers (n=54)	Non-shift workers (n=54)
pg/ml	*mean (SD); median*	*mean (SD); median*	*mean (SD); median*	*mean (SD); median*
IL-5	27.2 (67.0); 1.9	21.5 (47.3); 2.2	59.4 (85.9); 5.7	60.4 (123.1); 1.5
IL-13	10.6 (16.7); 5.0	9.0 (12.5); 3.1	22.5 (27.4); 4.1	20.3 (34.9); 1.4
IL-2	43.7 (74.5); 1.0	42.5 (68.9); 2.1	4.7 (17.6); 0.7	7.1 (21.3); 0.7
IL-6	121.2 (170.8); 46.2	115.8 (180.1); 47.7	181.2 (261.6); 58.7	191.2 (357.3); 45.5
IL-9	3.1 (6.4); 0.6	2.6 (6.6); 0.6	9.1 (14.0); 1.1	8.8 (16.3); 0.6
IL-10	4.5 (8.0); 1.3	6.1 (13.1); 0.6	6.3 (11.0); 0.9	8.8 (16.4); 0.6
IFNγ	511.5 (765.5); 116.1	485.4 (735.1); 153.4	879.6 (1356.0); 126.7	708.4 (1317.6); 41.1
TNFα	205.2 (348.3); 16.0	174.1 (285.9); 21.8	47.4 (82.3); 3.2	40.1 (73.2); 1.3
IL-17A	5.9 (13.7); 1.2	5.9 (13.1); 1.4	17.5 (25.4); 2.7	23.2 (48.3); 0.7
IL-17F	2.4 (7.5); 0.2	2.4 (6.8); 0.2	12.9 (20.9); 0.7	16.2 (38.6); 0.2
IL-4	0.7 (0.6); 0.5	0.9 (0.8); 0.7	0.6 (0.4); 0.5	0.7 (0.9); 0.4
IL-21	0.9 (0.3); 1.1	0.9 (0.3); 1.1	1.0 (0.3); 1.1	1.0 (0.6); 1.1
IL-22	4.0 (7.5); 1.4	3.8 (6.3); 1.6	5.6 (9.4); 2.3	5.6 (16.0); 0.5

SD, standard deviation.

* Statistically significant difference ($p<0.05$) between night shift workers and non-shift workers tested with the Mann-Whitney U test.
